# An Alternative Surgical Procedure for a Patient with Critically Restenosed and Kinked Carotid Artery: Graft Interposition

**DOI:** 10.1155/2011/572454

**Published:** 2011-10-24

**Authors:** Haydar Yaşa, Övünç Aslan, Barçın Özcem, Muhammet Akyuz, Ali Gürbüz, Galip Akhan

**Affiliations:** ^1^Department of Cardiovascular Surgery, Ataturk Training and Research Hospital, Izmir Katip Çelebi University, 35580 Izmir, Turkey; ^2^Department of Neurology, Ataturk Training and Research Hospital, Izmir Katip Çelebi University, 35580 Izmir, Turkey

## Abstract

According to the literature data, the prevalence of restenosis after carotid endarterectomy ranges between 6 and 36%. The etiological factor is intimal hyperplasia for early period, whereas it is atherosclerosis for late period. A 67-year-old male patient admitted to our clinic with a history of headache and minor stroke. His medical history was significant for right carotid endarterectomy 8 years ago. Recent Doppler ultrasound and digital substraction angiography revealed 75% stenosis and kinking corresponding to the segment distal to the endarterectomy region. Surgical endarterectomy is the treatment of choice in critical carotid stenosis. Endovascular therapy is primarily considered for patients if there is restenosis after carotid endarterectomy. However, the treatment modality is controversial in cases with concomitant carotid stenosis and kinking of internal carotid artery. We present our surgical approach to a case with significant stenosis and kinking of internal carotid artery. We performed a 6-mm-PTFE graft interposition between common and internal carotid artery and resection of the kinking segment.

## 1. Introduction

Restenosis after carotid endarterectomy is reported to have rates of 6–36% in various studies from two to fifteen-month follow-up periods [1, 2]. Etiologically intimal hyperplasia is responsible in early period, while atherosclerosis is responsible in late period.

Many factors play a role in the formation of atherosclerosis, but narrowing of the carotid artery due to kinking after endarterectomy is very rare in the literature.

For patients who develop restenosis after carotid endarterectomy, endovascular treatment is preferred, but patients who have restenosis with the presence of kinking in carotid artery are treated surgically.

Classic carotid endarterectomy has been performed more than 50 years ago [3]. Carotid endarterectomy performed for the first time in 1954, it still ranks first among the noncardiac vascular operations [4–6]. 

Eversion endarterectomy planned for the patient but severely ulcerated atherosclerotic plaque (invading the arterial wall) and very fragile arterial structure made us change our strategy peroperatively. Neither conventional endarterectomy nor eversion endarterectomy was suitable for the patient. In addition, because of the length of the excised segment end-to-end anastomosis could not be performed.

For this reason, synthetic graft (6 mm-ringed-polytetrafluoroethylene) interposition between the CCA (common carotid artery) and ICA (internal carotid artery) was performed.

## 2. Case Report

**Figure 1:**
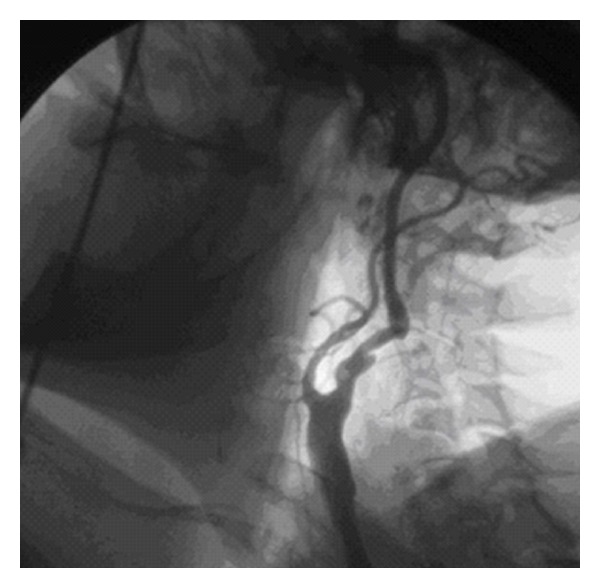
DSA view of the right carotid artery.

A sixty-seven-year-old male patient admitted to our clinic with complaints of headache and dizziness. Doppler ultrasound and digital subtraction angiography revealed 75% stenosis and kinking corresponding to the segment distal to the endarterectomy region. There was no severe stenosis in the contra lateral carotid artery. Minor stroke two months ago, the right carotid endarterectomy in 2002, and CABG in 1987 were reported in his medical history (Figure 1).

## 3. Surgical Technique

**Figure 2:**
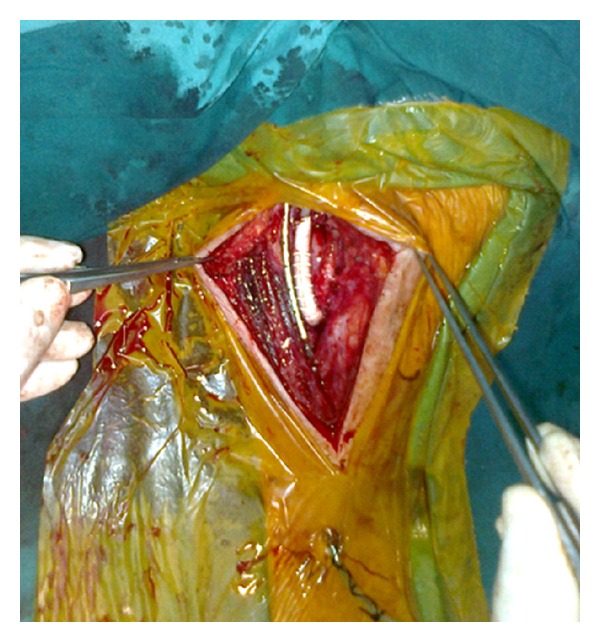
Intraoperative view of interposition graft.

The operation was performed under general anesthesia and intratracheal intubation. Supine position and neck hyperextension were achieved on the operating table. The incision made along the front edge of the sternocleidomastoid muscle. It was ended between the mastoid process and sternoclavicular junction. The incision was deepened and platsyma was passed. Sternocleidomastoid muscle was then ruled out. The internal jugular vein was deviated laterally and the common carotid artery was explored. Common carotid artery was mobilized, turned by a dissector, and hung up with silicon tape. Internal carotid artery was explored to the distal (nonplaque) soft portion, mobilized, turned by a dissector, and hung up with silicon tape. The same procedure was performed for the external carotid artery. While mobilizing the internal carotid artery, the 12th cranial nerve was also identified. The distal stump pressure was measured as 75 mmHg. The lesion in the internal carotid artery was in panarteritis form, and the arterial structure was very fragile. At the end of the lesion, severe elongation and fold were determined. 6-mm-ringed PTFE graft was anastomosed between common carotid artery and internal carotid artery. The proximal anastomosis was end-to-side and the distal anastomosis was end-to-end. ICA was 2.4 cm shortened and the kinking corrected (Figure 2). The patient discharged at the postoperative 5th day without any complication.

## 4. Discussion and Conclusion

In various studies, 6–36% restenosis was shown after carotid endarterectomy [1, 2].

This ratio is variable in different studies. Etiologically intimal hyperplasia is responsible in early period, while atherosclerosis is responsible in late period. In this case, inflammatory cells and atherosclerotic plaque formation was reported in pathological examination of the excised segment of the artery. Restenosis is seen more often in female. This is because of differences in platelet function and small vessel diameter in female. In several studies, the diameter of arteries in female reported narrower than male, but there was no difference between female with high body mass index and male. The internal carotid artery diameter under 5 mm and primary closure technique may cause restenosis in future, and under these conditions, patch plasty is a preferred technique.

Yet, in female patients primary closure technique instead of patch plasty increases the risk of restenosis [7].

Although there is no relationship in terms of restenosis between the gender and the patch used, there are some publications which indicate that more restenosis occurs in venous patch-used female patients.

In female, another factor causes restenosis is the kinking predisposition of vascular structure. Although this patient was a male folding and elongation was determined. In addition, primary closure in the first operation is believed to have a role in the presence of the folds. There is no consensus on application of intravascular shunt during carotid endarterectomy. While deciding the necessity of the use of shunt, some intraoperative monitoring techniques (the distal stump pressure, electroencephalogram (EEG), somatosensory evoked potential (SSEPI), cerebral oximetry, and awake neurological monitoring under limited sedation) are used.

Hafner et al. have reported 86% unnecessary use of shunts in patients with stump pressure below 50 mmHg. Shunt-related complications like shunt thrombosis embolization and intimal damage have also been reported [8, 9]. Therefore, we think that being more conservative in the use of shunt would be right. Intravascular shunt was not used in this case because the distal stump pressure was 75 mmHg. Another subject of discussion in cases developing restenosis is endovascular treatment. In recent years, the popularity of endovascular therapy has increased in the treatment of carotid artery stenosis [10]. Again, restenosis after carotid endarterectomy, contralateral internal carotid artery occlusion, cardiac problems, unstable neurological status, and surgical treatment impossible high-risk symptomatic patients (stenosis near the base of the head and neck radiotherapy) are reasons of preference for endovascular treatment. However, because of elongation and folding of carotid artery, surgical treatment is considered more appropriate in this case.

Primary or restenosis, there are alternative surgical techniques in treatment of critical carotid artery stenosis, which are conventional endarterectomy, eversion endarterectomy and graft interposition. End-to-end anastomosis is the preferred technique in patients with eversion endarterectomy. In this case, end-to-end anastomosis could not be performed due to the length of the excised segment. Therefore, graft interposition was performed. The use of autogenous material should be the first choice in suitable cases.

In this case, autologous graft could not be used because graft interposition was decided after cross-clamping. We think that graft interposition should be kept in mind as an alternative method in patients with critical internal carotid artery stenosis together with fold.
